# Drug repositioning with adaptive graph convolutional networks

**DOI:** 10.1093/bioinformatics/btad748

**Published:** 2023-12-09

**Authors:** Xinliang Sun, Xiao Jia, Zhangli Lu, Jing Tang, Min Li

**Affiliations:** School of Computer Science and Engineering, Central South University, Changsha, Hunan 410083, China; School of Computer Science and Engineering, Central South University, Changsha, Hunan 410083, China; School of Computer Science and Engineering, Central South University, Changsha, Hunan 410083, China; Research Program in Systems Oncology, Faculty of Medicine, University of Helsinki, FI00014 Helsinki, Finland; School of Computer Science and Engineering, Central South University, Changsha, Hunan 410083, China

## Abstract

**Motivation:**

Drug repositioning is an effective strategy to identify new indications for existing drugs, providing the quickest possible transition from bench to bedside. With the rapid development of deep learning, graph convolutional networks (GCNs) have been widely adopted for drug repositioning tasks. However, prior GCNs based methods exist limitations in deeply integrating node features and topological structures, which may hinder the capability of GCNs.

**Results:**

In this study, we propose an adaptive GCNs approach, termed AdaDR, for drug repositioning by deeply integrating node features and topological structures. Distinct from conventional graph convolution networks, AdaDR models interactive information between them with adaptive graph convolution operation, which enhances the expression of model. Concretely, AdaDR simultaneously extracts embeddings from node features and topological structures and then uses the attention mechanism to learn adaptive importance weights of the embeddings. Experimental results show that AdaDR achieves better performance than multiple baselines for drug repositioning. Moreover, in the case study, exploratory analyses are offered for finding novel drug–disease associations.

**Availability and implementation:**

The soure code of AdaDR is available at: https://github.com/xinliangSun/AdaDR.

## 1 Introduction

Computational drug repositioning is considered as an important alternative to the traditional drug discovery (Baker *et al.* 2018). It involves the use of de-risked compounds, with potentially lower overall development costs and shorter development timelines (Pushpakom *et al.* 2019). In other words, computational drug repositioning narrows down the search space for drug–disease associations by suggesting drug candidates for wet-lab validation. Hence, it has attracted remarkable attention. More importantly, some drugs have been successfully repositioned, bringing huge market and social benefits. For example, Sildenafil was initially employed as chest pain treatment when it was later discovered that it was a PDE5 inhibitor, which made Sildenafil a hit on the market.

In the past decades, machine learning-based approaches have gained considerable attention due to their high-quality prediction results in drug repositioning tasks. Most of these are data-driven methods that generally yield the latent feature from the known drug–disease interactive data, and then adopt various machine learning techniques to predict potential indications for a given drug. For example, Gottlieb *et al.* (2011) developed a computational approach called PREDICT to identify unknown drug–disease associations by integrating drug similarities and disease similarities. Moreover, Connectivity Map data (Lamb *et al.* 2006) is also employed in drug repositioning research. For instance, Iorio *et al.* (2010) used transcriptional responses to perform drug repositioning. However, feature-based machine learning methods heavily rely on the extraction of features and the selection of negative samples. With the development of high throughput technology and continuously updating databases, there are other types of biological entities frequently involved in drug–disease prediction, such as proteins, diseases, genes, and side effects. Therefore, network-based methods have been widely adopted. For example, Fiscon and Paci (2021) developed a network-based method named SAveRUNNER for drug repurposing, which offers a promising framework to efficiently detect putative novel indications for currently marketed drugs against diseases of interest. Wang *et al.* (2022) presented a novel scoring algorithm to repurpose drugs. Although the network-based methods have the advantage of good interpretability, their performances are not satisfactory (Luo *et al.* 2021).

To this end, a surge of more sophisticated techniques, such as matrix factorization and matrix completion approaches, have been applied to the drug repositioning tasks. In particular, matrix factorization and matrix completion techniques are of great popularity in drug repositioning tasks, due to their flexibility in integrating prior knowledge, and have shown promising results in application. In the constraint of bounded nuclear norm regularization, Yang *et al.* (2019) proposed BNNR method to complete the drug–disease matrix. To incorporate more prior knowledge, iDrug (Chen *et al.* 2020) was presented, which takes the drugs as the bridge to comprehensively utilize the target and disease information. Nevertheless, due to the high-complexity matrix operations, it is challenging to deploy matrix factorization and matrix completion approaches on large-scale datasets.

Recently, graph convolutional networks (GCNs) have achieved promising results in various tasks by utilizing both node features and graph topology. A few GCNs-based methods have been proposed for drug–disease association prediction. They generally formulate known drug–disease associations as a bipartite graph and then treat the drug repositioning problem as a link task. Besides, prior knowledge, e.g. drug–drug similarities and disease–disease similarities, is also used in their proposed models. For instance, Based on the heterogeneous information fusion strategy, Cai *et al.* (2021) design inter- and intra-domain feature extraction modules to learn the embedding of drugs and diseases. Considering the possible interactions between neighbors, Meng *et al.* (2022) presented a new weighted bilinear graph convolution operation to integrate the information of the known drug–disease association. Sun *et al.* (2022) considered the drug’s mechanism of action, and proposed an end-to-end partner-specific drug repositioning approach.

Although existing GCNs methods have achieved promising results in drug repositioning tasks, these methods have shortcomings in the following aspects. Firstly, they ignore the dependency between node features and topological structures to tasks, which limits their capabilities in distinguishing the contribution of components. Secondly, the proposed multi-source models based on GCNs heavily rely on the data sources. When some data are missing, the model performance will be decreased (Li *et al.* 2022). Despite these approaches can boost the model performance, their models still suffer from the bottleneck of data and are incapable of capturing the interactive information between topology and features. Therefore, directly applying the general GCNs framework on a drug–disease network inevitably restricts graph structure learning capability.

To tackle the above challenges, in this paper, we propose an adaptive GCN approach for drug repositioning. Inspired by the work (Wang *et al.* 2020), our key motivation is that the similarity between features and that inferred by topological structures are complementary to each other and can be fused adaptively to derive deeper correlation information. In order to fully exploit the information in feature space, we obtain the *k*-nearest neighbor graph generated from drug similarity features and disease similarity features as their feature structural graph, respectively. Taking the feature graph and the topology graph, we propagate the drug and disease features over both the topology space and feature space, so as to extract two embeddings in these two spaces. Considering common characteristics between the two spaces, we exploit the consistency constraint to extract embeddings shared by them. We further utilize the attention mechanism to automatically learn the importance weights for different embeddings.

In summary, the main contributions of this work are provided as the following:

- We propose a novel adaptive GCNs framework for drug repositioning tasks, which performs graph convolution operation over both topology and feature spaces.
- Considering the difference in topological structures and features, we adopt the attention mechanism to adequately fuse them, so as to distinguish the contribution to model results.
- Experimental results on the benchmark datasets clearly show that AdaDR outperforms the baseline models by a large margin in terms of AUPRC and demonstrates our proposed model’s utility in drug repositioning tasks.

## 2 Materials and methods

**Figure 1.**
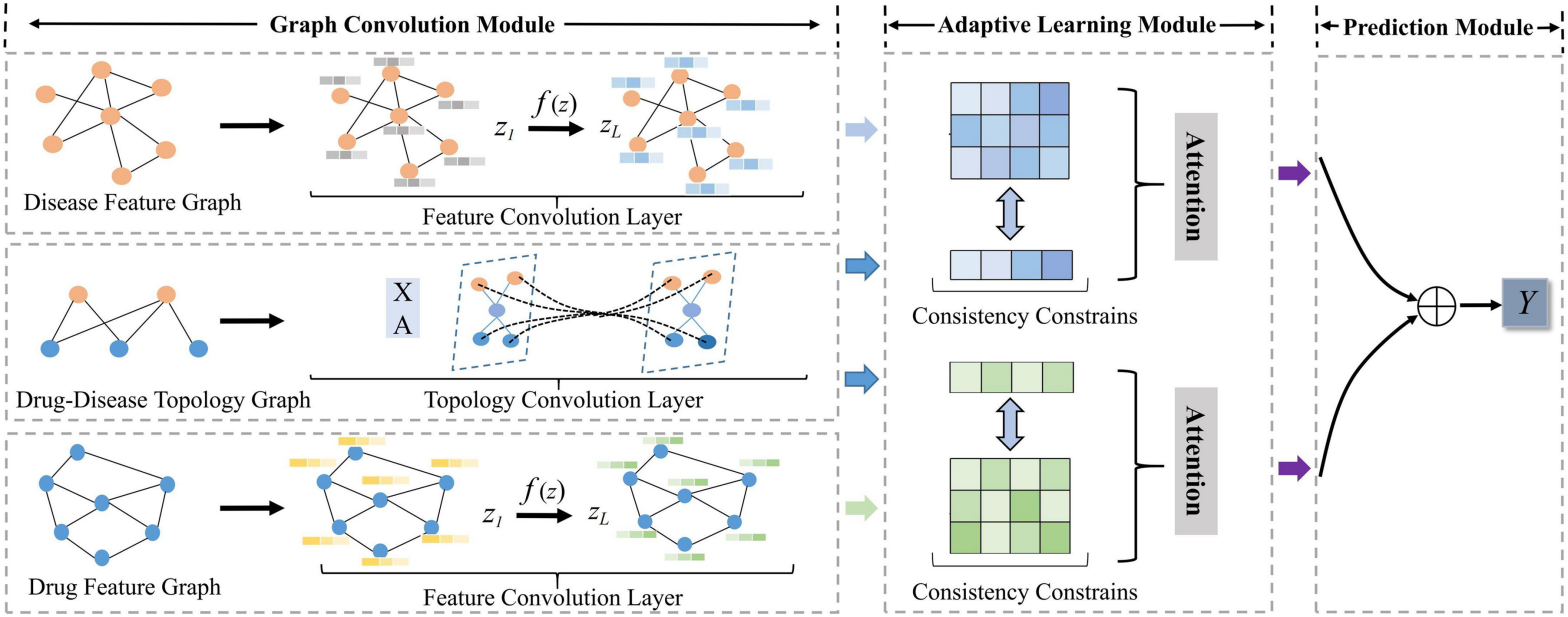
The overall framework of AdaDR consists of three parts: (i) graph convolution module to represent the drug/disease embeddings in feature and topology space; (ii) adaptive learning module with attention mechanism to distinguish the importance of obtained embeddings. Besides, the consistency constraint is used to push closer the embeddings in different spaces in this module; (iii) prediction module to concatenate embeddings as the output to predict results.

In this section, we first describe the benchmark dataset used in the proposed model. We then introduce the AdaDR model framework, which mainly comprises three components. As the Fig. 1 depicts, (i) graph convolution module which contains the feature convolution layer and the topology convolution layer to represent the graph embeddings. (ii) Adaptive learning module to distinguish the importance of obtained embeddings by utilizing attention mechanism. Besides, in this module, the common semantics information between feature and topology space is extracted with the consistency constraint. (iii) Finally, prediction module to concatenate embeddings as the output to predict results.

### 2.1 Datasets

To comprehensively evaluate the proposed model performance, we exploit four benchmark datasets, e.g. Gdataset (Gottlieb *et al.* 2011), Cdataset (Luo *et al.* 2016), Ldataset (Yu *et al.* 2021), and LRSSL (Liang *et al.* 2017), which are widely used in drug repositioning tasks. The Gdataset is also treated as the gold standard dataset, which includes 1933 proven drug–disease associations between 593 drugs taken from DrugBank and 313 diseases listed in the OMIM database. Cdataset contains 663 drugs, 409 diseases and 2352 interacting drug–disease pairs, which first appears in Luo *et al.* (2016) study. Ldataset is compiled by CTD dataset (Davis *et al.* 2017), which includes 18 416 associations between 269 drugs and 598 diseases. The last dataset LRSSL contains 3051 validated drug–disease associations involving 763 drugs and 681 diseases. Meanwhile, to construct the drug/disease feature graph, we also utilize the similarity features for drugs and diseases. It is worth noting that different similarity feature profiles can produce different results for the model. According to the previous studies (Yu *et al.* 2021, Meng *et al.* 2022), we use drug similarity based on chemical substructures and the semantic similarity of disease phenotypes to construct the drug/disease feature graph to obtain the best performance for model. Specifically, the similarity score between drugs is calculated by their corresponding 2D chemical fingerprints. The data statistics are briefly shown in Table 1.

**Table 1.** Statistics of the four benchmark datasets.

Dataset	No. of drugs	No. of diseases	No. of associations	Sparsity
Gdataset	593	313	1933	0.0104
Cdataset	663	409	2532	0.0093
LRSSL	763	681	3051	0.0059
Ldataset	269	598	18 416	0.1145

### 2.2 Feature convolution layer

In order to capture the underlying structure of drugs and diseases in feature space, we construct a *k*-nearest neighbor graph (*k*NN) based on their similarity matrix, respectively. Here, we denote the drug similarity matrix by $X^{r}\in R^{n\times n}$, where *n* is the number of drugs. The adjacency matrix of drug *k*NN graph is represented by the binary matrix $A^{r}\in R^{n\times n}$, where each entry of $A^{r}$ is constructed based on the similarity of each pair of drugs. The entry $A_{ij}^{r}$ of $A^{r}$ is defined as:

(1) $$A_{ij}^{r}=\{\begin{array}{c} 1,\;\text{if}\;r_{j}\in {\tilde{N}}_{k}\left(r_{i}\right)\\ 0,\;\text{otherwise}\; \end{array}$$

where ${\tilde{N}}_{k}\left(r_{i}\right)=\left\{r_{i}\right\}\cup N_{k}\left(r_{i}\right)$ is a set of *r_i_*’s extended *k*-nearest neighbors including *r_i_*, and $N_{k}\left(r_{i}\right)$ is the *k*-nearest neighbors of drug *r_i_*. In the same way, we denote the disease similarity matrix by $X^{d}\in R^{m\times m}$, where *m* is the number of diseases. The entry $A_{ij}^{d}$ of matrix $A^{d}$ is defined as:

(2) $$A_{ij}^{d}=\{\begin{array}{c} 1,\;\text{if}\;d_{j}\in {\tilde{N}}_{k}\left(d_{i}\right)\\ 0,\;\text{otherwise}\; \end{array}$$

where ${\tilde{N}}_{k}\left(d_{i}\right)=\left\{d_{i}\right\}\cup N_{k}\left(d_{i}\right)$ is a set of *d_i_*’s extended *k*-nearest neighbors including *r_i_*, and $N_{k}\left(d_{i}\right)$ is the *k*-nearest neighbors of drug *d_i_*.

With the drug *k*NN graph $G_{r}=(A^{r},X^{r})$ and the disease *k*NN graph $G_{d}=(A^{d},X^{d})$ in feature space, we utilize the typical GCN (Kipf and Welling 2017) to represent constructed graphs’ *l*th layer output:

(3) $$\{\begin{array}{c} Z_{r}^{\left(l\right)}=\mathrm{ReLU}\left(D_{r}^{-\frac{1}{2}}A^{r}\;D_{r}^{-\frac{1}{2}}Z_{r}^{\left(l-1\right)}W_{r}^{\left(l\right)}\right)\\ Z_{d}^{\left(l\right)}=\mathrm{ReLU}\left(D_{d}^{-\frac{1}{2}}A^{d}\;D_{d}^{-\frac{1}{2}}Z_{d}^{\left(l-1\right)}W_{d}^{\left(l\right)}\right) \end{array}$$

where $Z_{r}^{\left(l\right)},Z_{d}^{\left(l\right)}\in R^{n}$ are the *k*th layer’s information propagated for drugs and diseases, respectively; $W_{r}^{\left(l\right)},W_{d}^{\left(l\right)}$ are the weight matrices of the *l*th layer in GCN. ReLU denotes the Relu activation function and the initial $Z_{r}^{\left(0\right)}=X^{r},Z_{d}^{\left(0\right)}=X^{d}$; $D_{r},D_{d}$ represent the diagonal degree matrix of $A^{r}$ and $A^{d}$, respectively. We denote the drug and disease last layer output embedding as $Z_{Fr}$ and $Z_{Fd}$, respectively. In this way, we can learn the embedding which captures the specific information in feature space.

### 2.3 Topology convolution layer

As for the topology space, we take the known drug–disease associations as the input graph. Specifically, we build a GCMC (Berg *et al.* 2018) as the backbone to obtain the drug–disease representations of drugs and diseases.

In our scenario, the known and unknown drug–disease associations are treated as different edge type and assigned separate processing channels for each edge type t∈{0,1}. To be specific, each edge type of graph convolution can be seen as a form of message passing, where vector-valued messages are being passed and transformed across the edges of the graph. In our model, we assign a specific transformation for each edge type, resulting in edge-type specific messages $\mu_{j\to i,t}$, from diseases(*d*) *j* to drugs(*r*) *i* of the following form:

(4) $$MP(\mu_{j\to i,t})=\frac{1}{c_{ij}}W_{t}x_{j}$$

where *c_ij_* is a symmetric normalization constant $\sqrt{\left|N(r_{i})||N(d_{j})\right|}$, with $N(r_{i})$ denoting the set of neighbors of drug node *i* and $N(d_{j})$ denoting the set of neighbors of disease node *j*. $W_{t}$ is an edge-type specific parameter matrix and *x_j_* is the feature vector of disease node *j*. Messages $MP(\mu_{i\to j,t})$ from drugs to diseases are processed in an analogous way. After the message passing step, we can accumulate incoming messages at every node by summing over all neighbors $N_{t\in \{0,1\}}(r_{i})$ connected by a specific edge-type, and by accumulating the results for each edge type into a single vector representation:

(5) $$h_{i}=\sigma \left[\mathrm{sum}\left(\sum_{j\in N_{t\in \{0,1\}}\left(r_{i}\right)}MP(\mu_{j\to i,t})\right)\right]$$

where sum denotes an accumulation operation; *σ* denotes an activation function such as the tanh. To obtain the final representation of drugs, we transform the intermediate output $z_{i}$ by a linear operator:

(6) $$z_{i}=Wh_{i}$$

The disease embedding $z_{j}$ is computed analogously. Note that, in the linear operator, the parameter matrix **W** of drug nodes is the same as that of disease nodes, because the model is trained without side information of the nodes. By applying the above transformation to all nodes in the drug–disease graph, we can obtain the final representation of drugs $Z_{Tr}$ and diseases $Z_{Td}$ in the topology space.

### 2.4 Attention mechanism for adaptive learning

Now we obtain specific drug embeddings $Z_{Fr}$ and $Z_{Tr}$, and specific disease embeddings $Z_{Fd}$ and $Z_{Td}$ in feature space and topology space, respectively. Considering the prediction result can be correlated with them, we use the attention mechanism to adaptively learn the corresponding importance of drug embeddings and disease embeddings as follows:

(7) $$\begin{array}{c} \left(\alpha_{fr},\alpha_{tr}\right)=\mathrm{att}\left(Z_{Fr},Z_{Tr}\right)\\ \left(\alpha_{fd},\alpha_{td}\right)=\mathrm{att}\left(Z_{Fd},Z_{Td}\right) \end{array}$$

here att is a neural network which performs the attention operation. $\alpha_{fr},\;\alpha_{tr}\in R^{n\times 1}$ and $\alpha_{fd},\;\alpha_{td}\in R^{m\times 1}$ indicate the attention values of drug nodes and disease nodes with embeddings $Z_{Fr},Z_{Tr}$ and $Z_{Fd},Z_{Td}$, respectively.

Specifically, taking the $z_{Fr}^{i}\in R^{1\times h}$, that is, the *i*th row of $Z_{Fr}$, as an example, we transform the embedding through a nonlinear transformation. After one shared attention vector $q\in R^{h^{\prime }\times 1}$ is used to get the attention value $\omega_{Fr}^{i}$ as follows:

(8) $$\omega_{Fr}^{i}=q^{T}\cdot \tanh\left(W_{Fr}\cdot {\left(z_{Fr}^{i}\right)}^{T}+b_{Fr}\right)$$

where $W_{Fr}\in R^{h^{\prime }\times h}$ is the weight matrix and $b_{Fr}\in R^{h^{\prime }\times 1}$ is the bias vector for embedding matrix $Z_{Fr}$. Similarly, we can get the attention values $\omega_{Tr}^{i}$ for drug node *i* in embedding matrices $Z_{Tr}$. With the analogous way, for *j*th disease node, we can get $\omega_{Fd}^{j}$ and $\omega_{Td}^{j}$ from $Z_{Fd}$ and $Z_{Td}$, respectively. We then normalize the attention values with softmax function to get the final drug weight and disease weight:

(9) $$\begin{array}{c} \alpha_{Fr}^{i}=\mathrm{softmax}\left(\omega_{Fr}^{i}\right)=\frac{\;\text{exp}\;\left(\omega_{Fr}^{i}\right)}{\;\text{exp}\;\left(\omega_{Fr}^{i}\right)+\text{exp}\;\left(\omega_{Tr}^{i}\right)}\\ \alpha_{Fd}^{j}=\mathrm{softmax}\left(\omega_{Fd}^{j}\right)=\frac{\;\text{exp}\;\left(\omega_{Fd}^{j}\right)}{\;\text{exp}\;\left(\omega_{Fd}^{j}\right)+\text{exp}\;\left(\omega_{Td}^{j}\right)} \end{array}$$

Similarly, $\alpha_{Tr}^{i}=\mathrm{softmax}\left(\omega_{Tr}^{i}\right)$ and $\alpha_{Td}^{j}=\mathrm{softmax}\left(\omega_{Td}^{j}\right)$. For all the *n* drug nodes and *m* disease nodes, we obtain the learned weights $\alpha_{fr}=[\alpha_{Fr}^{i}],\;\alpha_{tr}=[\alpha_{Tr}^{i}]\in R^{n\times 1}$ and $\alpha_{fd}=[\alpha_{Fd}^{j}],\;\alpha_{td}=[\alpha_{Td}^{j}]\in R^{m\times 1}$, and denote $\alpha_{Fr}=\mathrm{diag}(\alpha_{fr}),\alpha_{Tr}=\mathrm{diag}(\alpha_{tr})$ and $\alpha_{Fd}=\mathrm{diag}(\alpha_{fd}),\alpha_{Td}=\mathrm{diag}(\alpha_{td})$. Then we combine these embeddings to obtain the final drug embedding $Z_{r}$ and disease embedding $Z_{d}$:

(10) $$\begin{array}{c} Z_{r}=\alpha_{Fr}\cdot Z_{Fr}+\alpha_{Tr}\cdot Z_{Tr}\\ Z_{d}=\alpha_{Fd}\cdot Z_{Fd}+\alpha_{Td}\cdot Z_{Td} \end{array}$$

### 2.5 Prediction and optimization

To obtain the final prediction result, we concatenate two obtained embeddings to represent the drug–disease pair. Particularly, we utilize a three-layer MLP neural network to represent ${\hat{y}}_{ij}$, that is, how likely it is that the drug can be indicated for the disease:

(11) $${\hat{y}}_{ij}=\mathrm{MLP}\;\left(z_{r}^{i}||z_{d}^{j}\right)$$

The binary cross-entropy (BCE) loss is used as the main loss:

(12) $$L_{\mathrm{bce}}=\underset{\left(i,j\right)}{-\sum }y_{ij}\cdot \;\text{log}\;\left({\hat{y}}_{ij}\right)+\left(1-y_{ij}\right)\cdot \;\text{log}\;\left(1-{\hat{y}}_{ij}\right)$$

here (*i*, *j*) denotes the pair for drug *i* and disease *j*; *y_ij_* is the truth label. Considering the common semantics between feature space and topology space, we exploit a consistency constraint to enhance their commonality. For drug embeddings, we use *L*_2_-normalization to normalize the embedding matrix. Then, the two normalized matrix can be utilized to capture the similarity of *n* drug nodes in different spaces as $S_{F}^{r}$ and $S_{T}^{r}$ as follows:

(13) $$\begin{array}{c} S_{F}^{r}=Z_{Fr}\cdot Z_{Fr}^{T}\\ S_{T}^{r}=Z_{Tr}\cdot Z_{Tr}^{T} \end{array}$$

Therefore, we can give rise to the following constraint:

(14) $$L_{Cr}={\left\|S_{F}^{r}-S_{T}^{r}\right\|}_{F}^{2}$$

In the same way, the disease embedding constraint $L_{Cd}$ is caculated. We achieve the final loss L by weighted combing the BCE loss $L_{\mathrm{bce}}$, the consistency constraint $L_{Cr}$ and $L_{Cd}$.

(15) $$L=L_{\mathrm{bce}}+\lambda L_{Cr}+\lambda L_{Cd}$$

where *λ* is the hyperparameter to balance the three terms.

### 2.6 Model discussion

By integrating the different space information of the same drug/disease into model can provides the rich semantic for drug/disease representation. Consequently, the combined prediction of the two spaces can be further boosted. Neverthless, when an adaptive graph neural network method is used to predict a drug–disease prediction problem, some questions must be answered. Whether it is appropriate to exploit isomorphic graph and heterogeneous graph to extract embeddings together. In our model, the basic assumption is that the similarity between features and that inferred by topological structures are complementary to each other. In other words, the constructed drug/disease feature graph and the known drug–disease association topology graph should be approximate. However, the known drug–disease association topology graph is a bipartite graph in which a drug directly links a disease, while a drug/disease directly links a drug/disease in the drug/disease feature graph. That is, the graph information derived from the constructed drug feature graph and disease feature graph will conflict with the known drug–disease association bipartite graph information. Therefore, this graph learning mechanism will make some confusion about the proposed model.

**Figure 2.**
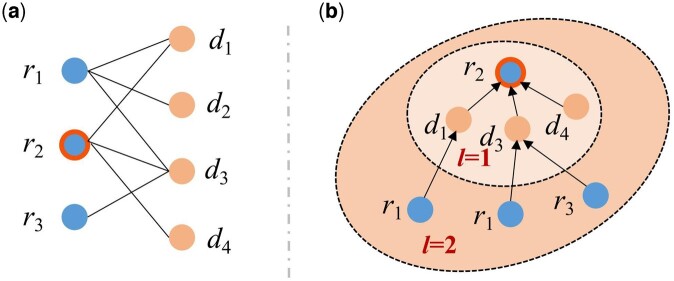
An illustration of the drug–disease high-order connectivity. (a) is the known drug–disease association bipartite graph; (b) depicts the high-order connectivity with tree structure. The node *r*_1_ labeled with the double circle is the target drug to treat diseases.

To shed more light on graph patterns learning in adaptive GCNs models, we provide an illustration as shown in Fig. 2. It illustrates the concept of high-order connectivity. The target drug is *r*_2_, labeled with the double circle in the left subfigure of drug–disease association graph. The right subfigure shows the tree structure that is expanded from *r*_2_. The high-order connectivity denotes the path that reaches *r*_2_ from any node with the path length *l* larger than 1. In this sense, we demonstrate that when the path length *l* gets an even number, the drugs still link drugs in the drug–disease bipartite graph. In the analogous way, the same conclusion can be drawn from diseases. Consequently, under the path of even number length, the odd hop connected nodes practically act as a bridge to make the target drug/disease node still links the same type of nodes. To this end, we empirically adopt two layers of convolution in the topology convolution module, since deeper layers can result in bad generalization performance. To sum up, the basic assumption that the constructed drug/disease feature graph and the known drug–disease association topology graph should be approximate still can be supported.

## 3 Results and discussion

### 3.1 Parameter setting

There are several hyperparameters in AdaDR such as the total training epoch *α*, the learning rate *lr*, the regular dropout rate *γ*, the number of neighbors *K* in the feature graph and the trade-off parameter *λ*. We consider different combinations of these parameters from the ranges α∈{1000,2000,3000,4000}, lr∈{0.001,0.01,0.1}, γ∈{0.1,0.2,0.3,0.4}. By adjusting the parameters empirically, we set the parameter *α* = 4000, *lr *=* *0.01 and γ=0.3 for AdaDR in all experiments. For parameters, i.e. *K* and *λ*, the detailed tuning process is described in the 3.5 part. Besides, the parameters in the compared approaches are set to the default values on their papers.

### 3.2 Baseline model

To evaluate the performance of our proposed model, we compare AdaDR with the seven state-of-the-art drug repositioning methods listed below. The baseline model contains three GCNs based models (e.g. DRHGCN, NIMGGCN, DRWBNCF) and three matrix completion based models (e.g. MBiRW, iDrug, BNNR). To evaluate the performance of our proposed model, we compare AdaDR with the seven state-of-the-art drug repositioning methods listed below. The baseline model contains four GCNs based models and three matrix completion based models.

- MBiRW (Luo *et al.* 2016) is a bi-random walk algorithm, which uses sparse drug–disease associations to enhance the similarity measures of drug and disease to perform association prediction.
- iDrug (Chen *et al.* 2020) is a matrix completion based method, which utilizes the cross-network drug-related information to achieve better model performance.
- BNNR (Yang *et al.* 2019) completes the drug–disease matrix under the low-rank assumption, which integrates the drug–drug, disease–disease and drug–disease information.
- DRHGCN (Cai *et al.* 2021) fuses the inter- and intra-domain embeddings to enhance the representation of drug and disease.
- NIMCGCN (Li *et al.* 2020) is a variant induction matrix completion method. It is widely used to predict drug–disease associations.
- DRWBNCF (Meng *et al.* 2022) models the complex drug–disease associations based on weighted bilinear neural collaborative filtering approach.

### 3.3 Performance of AdaDR in cross-validation

We execute 10-fold cross-validation to evaluate the performance of AdaDR. During the 10-fold cross-validation, all known and unknown drug–disease associations are randomly divided into 10 exclusive subsets of approximately equal size, respectively. Each subset is treated as the testing set in turn, while the remaining nine subsets are used as the training set. Then, the area under the receiver operating characteristic curve (AUROC) and the area under the precision-recall curve (AUPRC) are adopted to measure the overall performance of AdaDR. It should be noted that AUPRC is often more informative than AUROC when the data has class imbalance problem (Davis and Goadrich 2006, Saito and Rehmsmeier 2015). Therefore, in our experimental scenario, we pay more attention to the performance of the model AUPRC. Moreover, to relieve the potential data bias of cross-validation, we repeat 10 times 10-fold cross-validation for AdaDR and other models and calculate the average value and standard deviation of the results. The results of four benchmark datasets are shown in Table 2.

**Table 2.** The AUROC and AUPRC are obtain under the 10 times 10-fold cross-validation on Gdataset, Cdataset, LRSSL, and Ldataset.^a^

Dataset	MBiRW	iDrug	BNNR	DRHGCN	NIMCGCN	DRWBNCF	AdaDR
AUROC							
Gdataset	0.896 ± 0.014	0.905 ± 0.019	0.937 ± 0.010	0.948 ± 0.011	0.821 ± 0.011	0.923 ± 0.013	0.952 ± 0.006
Cdataset	0.920 ± 0.008	0.926 ± 0.010	0.952 ± 0.010	0.964 ± 0.005	0.827 ± 0.017	0.941 ± 0.011	0.966 ± 0.006
LRSSL	0.893 ± 0.015	0.900 ± 0.008	0.922 ± 0.012	0.961 ± 0.006	0.777 ± 0.012	0.935 ± 0.011	0.950 ± 0.010
Ldataset	0.765 ± 0.007	0.838 ± 0.005	0.866 ± 0.004	0.851 ± 0.007	0.843 ± 0.001	0.824 ± 0.005	0.881 ± 0.003
Average.★	0.868	0.892	0.919	0.931	0.817	0.906	0.937
AUPRC							
Gdataset	0.106 ± 0.019	0.167 ± 0.027	0.328 ± 0.029	0.490 ± 0.041	0.123 ± 0.028	0.484 ± 0.027	0.588 ± 0.041
Cdataset	0.161 ± 0.019	0.250 ± 0.027	0.431 ± 0.020	0.580 ± 0.035	0.174 ± 0.071	0.559 ± 0.021	0.671 ± 0.030
LRSSL	0.030 ± 0.004	0.070 ± 0.009	0.226 ± 0.021	0.384 ± 0.022	0.087 ± 0.010	0.349 ± 0.034	0.475 ± 0.042
Ldataset	0.032 ± 0.003	0.086 ± 0.004	0.142 ± 0.007	0.498 ± 0.012	0.117 ± 0.002	0.419 ± 0.006	0.569 ± 0.009
Average.★	0.082	0.143	0.282	0.488	0.125	0.453	0.576

a Average.

★ shows the average AUROC/AUPRC over four datasets and the best result in each row is underline.

Based on the results, we can first see that the final average results over four datasets obtained by AdaDR outperform all comparison methods in 10 times 10-fold cross-validation due to the feature integration capacity. For instance, we observe that AdaDR achieves the final average AUROC value of 0.937, which is 0.6% higher than the second-best method DRHGCN, and the average AUPRC obtained by AdaDR is 0.576, which is 8.8% higher than that obtained by the second-best method DRHGCN. It is worth noting that AdaDR achieves the highest AUPRC over three datasets (i.e. Gdataset, Cdataset and Ldataset) and obtains the second-best AUROC on the LRSSL dataset, which is lower than the best method DRHGCN. Meanwhile, compared with GCNs based methods, e.g. DRHGCN, NIMCGCN and DRWBNCF, AdaDR is superior to them in terms of average results because of its strong ability to integrate topology and features. Most importantly, it is obvious that our AdaDR significantly surpasses other methods by a large margin on four benchmarks under AUPRC metrics. For example, our results are 9.8%, 9.1%, 9.1%, and 7.1% higher than that of the second-best method DRHGCN in terms of AUPRC on Gdataset, Cdataset, LRSSL and Ldataset, respectively. The above results can well demonstrate the effectiveness of our proposed method.

### 3.4 Predicting indications for new drugs

**Figure 3.**
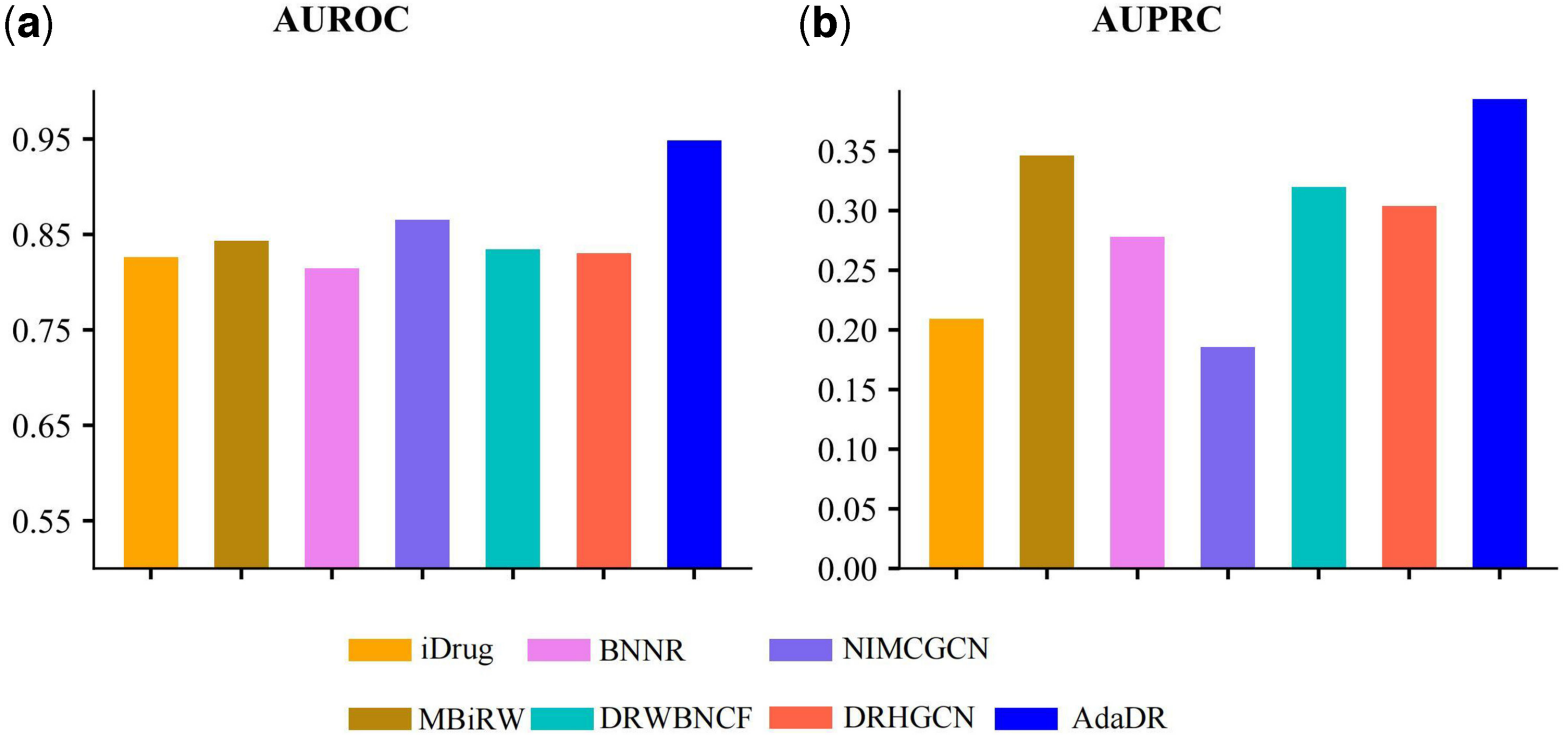
The performance of all methods in predicting potential diseases for new drugs on the Gdataset. (a) The AUROC of prediction results obtained by applying AdaDR and other competitive methods. (b) The AUPRC of prediction results obtained by applying AdaDR and other competitive methods.

The newly predicted drug–disease associations can aid in drug repositioning. To this end, we conduct a new experiment to evaluate the capability of AdaDR for predicting potential indications for new drugs. Specifically, for each drug *r_i_*, we delete all known drug–disease associations about drug *r_i_* as the testing set and use all the remaining associations as the training samples. It should be noted that Gdataset is also known as the gold standard dataset which collects comprehensive associations from multiple data sources. Thus, we use Gdataset to evaluate the model performance and calculate the average of all test results. In total, we test 593 drugs and perform the experiment by once. The results of Gdataset are shown in Fig. 3. Compared with the seven other methods, AdaDR achieves the top performance. In terms of AUROC, as shown in Fig. 3a, we observe that AdaDR achieves an AUROC value of 0.948, which is better than that of the other methods. Meanwhile, as shown in Fig. 3b, AdaDR achieves an AUPRC of 0.393, which are higher than all the other approaches.

### 3.5 Parameter analysis

**Figure 4.**
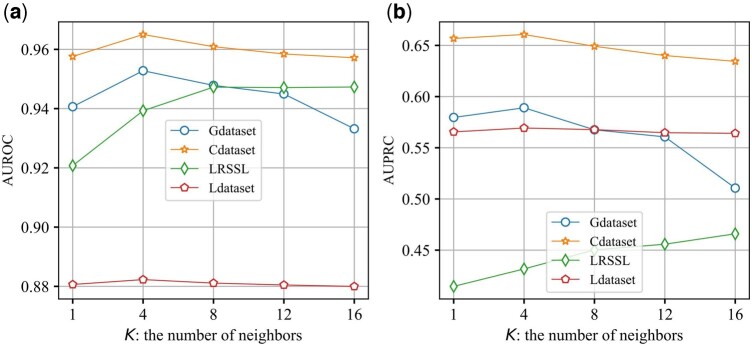
Effect of different neighbor numbers on the performance of AdaDR. (a) The variation of AUROC. (b) The variationof AUPRC.

We further make the experimental verification about the impact of the trade-off parameter *λ* and the number of neighbors in the feature graph on all datasets. The number of neighbors *K* in the feature graph is crucial for model performance, we analyze the stabilities of AdaDR on all datasets by varying *K*. The results about the impact of the number of neighbors are shown in Fig. 4. Intuitively, we vary *K* value in range of [1,4,8,12,16]. As we can see, for Gdataset, Cdataset and Ldataset, the number of neighbors in feature space is set as *K *=* *4, AdaDR achieves the best results. Another interesting results can observe that, for LRSSL, as the number of *K* increases, the results of AdaDR generally improves. This is because that LRSSL is very sparse. When the number of neighbors increases, more information in feature space will be incorporated.

**Figure 5.**
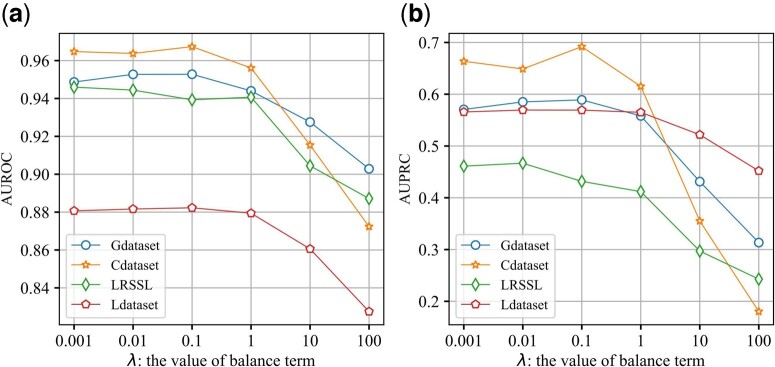
Effect of different *λ* values on the performance of AdaDR. (a) The variation of AUROC. (b) The variation of AUPRC.

Trade-off parameter *λ* is introduced to appropriately weigh BCE loss and consistency constraint loss. We let the trade-off value *λ* vary from [0.001,0.01,0.1,1,10,100] for all datasets. Figure 5 shows the variation of AUROC and AUPR with different *λ*. It can be seen that, for Gdataset, Cdataset, and Ldataset, when the values of *λ* are 0.1, the optimal AUROC and AUPR performance are obtained. Therefore, we set λ=0.1 on the above three datasets as the model parameter. For LRSSL, we can observe that AdaDR gets satisfactory AUROC and AUPR when the trade-off value is set λ=0.1 and λ=0.01, respectively. Finally, for LRSSL, the trade-off value is selected as λ=0.01 in our model due to the unbalance of positive and negative samples.

### 3.6 Ablation study

In this section, we compare different strategies for training our AdaDR on all datasets to investigate their effectiveness. For training an adaptive GCNs, we analyze the following four cases:

- **AdaDR-w/o-l**: AdaDR without constraint $L_{Cr}$ and $L_{Cd}$.
- **AdaDR-w/o-a**: AdaDR without attention mechanism.
- **AdaDR-w/o-f**: AdaDR without using the feature space information.
- **AdaDR-w/o-t**: AdaDR without using the topology space information.

Table 3 reports the results of different strategies for training AdaDR. It clearly demonstrates that each kind of strategy of AdaDR can improve the model performance, especially after using drug/disease topology features in the adaptive GCNs. Moreover, we mainly make the following four observations: (i) The topology space information is the most important component. Because it directly contains drug–disease association information which helps the model to learn the potential drug–disease association pattern. Thus, compared with other training strategies, it has the most significant improvement in model performance. (ii) The feature space information benefits the model. Without the feature space information, the model is only learned with topology space information and therefore fails to sufficiently exploit data information. (iii) Removing the consistency constraint from the AdaDR will decrease the performance. This is due to the fact that the consistency constraint improves the generality of the representations and thus benefits the learning of the model. (iv) The attention mechanism can better encode topology space information and feature space information. When removing the attention mechanism from the AdaDR, the model performance will decrease. The above observations verify the effectiveness and importance of each component in the AdaDR.

**Table 3.** The AUROC and AUPRC of models corresponding to the different training strategies on all datasets.

Method	Gdataset	Cdataset	LRSSL	Ldataset
AUROC				
AdaDR-w/o-l	0.949 ± 0.005	0.964 ± 0.005	0.951 ± 0.009	0.881 ± 0.004
AdaDR-w/o-a	0.949 ± 0.010	0.964 ± 0.004	0.945 ± 0.010	0.879 ± 0.003
AdaDR-w/o-f	0.943 ± 0.006	0.958 ± 0.006	0.937 ± 0.012	0.878 ± 0.003
AdaDR-w/o-t	0.908 ± 0.008	0.936 ± 0.012	0.899 ± 0.013	0.805 ± 0.010
AdaDR	0.952 ± 0.006	0.966 ± 0.006	0.950 ± 0.010	0.881 ± 0.003
AUPRC				
AdaDR-w/o-l	0.576 ± 0.048	0.659 ± 0.025	0.470 ± 0.044	0.568 ± 0.009
AdaDR-w/o-a	0.569 ± 0.045	0.657 ± 0.032	0.454 ± 0.036	0.564 ± 0.008
AdaDR-w/o-f	0.564 ± 0.043	0.632 ± 0.033	0.445 ± 0.042	0.563 ± 0.010
AdaDR-w/o-t	0.324 ± 0.045	0.439 ± 0.043	0.294 ± 0.041	0.398 ± 0.004
AdaDR	0.588 ± 0.041	0.671 ± 0.030	0.475 ± 0.042	0.569 ± 0.009

### 3.7 Analysis of attention mechanism

**Figure 6.**
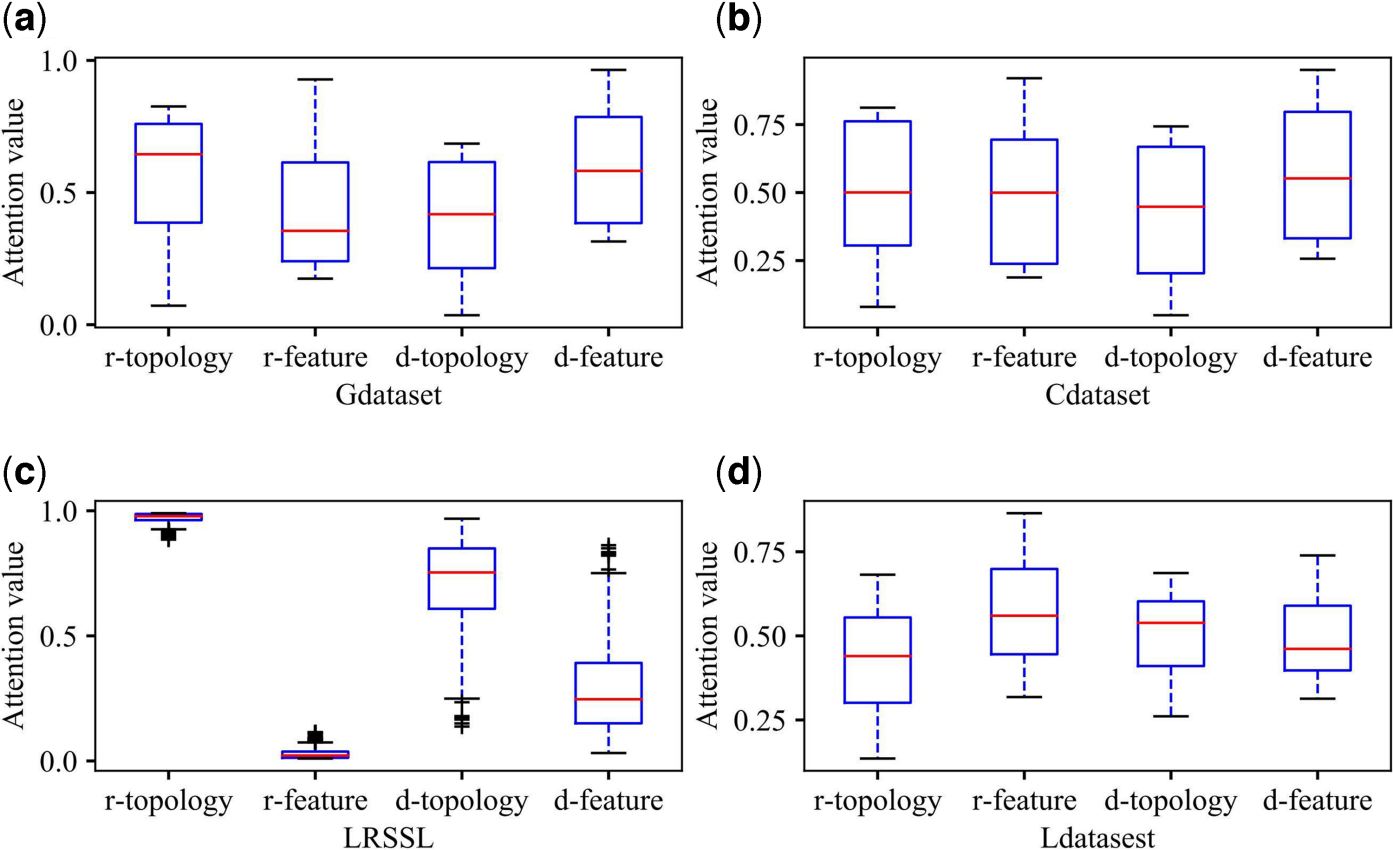
Analysis of attention distribution. r-topology and r-feature denote the drug topology attention value and drug feature attention value, respectively. d-topology and d-feature denote the disease topology attention value and disease feature attention value, respectively. (a), (b), (c) and (d) represent the results of attention values for Gdataset, Cdataset, Ldataset and LRSSL, respectively.

In order to investigate whether the attention values learned by AdaDR are meaningful, we analyze the attention distribution. Our proposed model learns two specific drug and two specific disease embeddings, each of which is associated with the attention values. We conduct the attention distribution analysis on all datasets, where the results are shown in Fig. 6. As we can see, for Gdataset, Cdataset, LRSSL, the attention values of drug specific embeddings in topology space are larger than the values in feature space. Besides, we find that the attention values of drug specific embeddings in feature space are larger than the values in topology space on Ldataset. This implies that the information in topology space should be more important than the information in feature space. For specific disease embeddings, on Gdataset and Cdataset, the attention values of disease specific embeddings in feature space are larger than the values in topology space. Conversely, on LRSSL and Ldataset, the attention values of disease specific embeddings in topology space are larger than the values in feature space. In summary, the experiment demonstrates that our proposed AdaDR is able to adaptively assign larger attention values for more important information.

### 3.8 Case studies

We conduct two case studies to further verify AdaDR by performing a literature-based evaluation of new hits. Specifically, we apply AdaDR to predict candidate drugs for two diseases including Alzheimer’s disease (AD) and Breast carcinoma (BRCA). AD is a progressive neurological degenerative disease that has no efficacious medications available yet. BRCA is a phenomenon in which breast epithelial cells proliferate out of control under the action of a variety of oncogenic factors. Although there are many drugs for breast cancer, such as Paclitaxel, Carboplatin and so on, a wider choice of drugs may provide better treatment options.

During the process, all the known drug–disease associations in the Gdataset are treated as the training set and the missing drug–disease associations regarded as the candidate set. After all the missing drug–disease associations are predicted, we subsequently rank the candidate drugs by the predicted probabilities for each drug. We focus on the top five potential drugs for breast carcinoma and AD and adopt highly reliable sources (i.e. CTD and PubMed) to check the predicted drug–disease associations. Table 4 reports candidate drugs with evidence. For AD and breast carcinoma, we can see that among the top five drugs ranked according to their predicted scores have been validated by various evidence from authoritative sources and literature (100% success rate). Moreover, our model can make interpretable results. Taking Paclitaxel as an example, our model predict it can treat breast cancer. This is indeed supported by authoritative sources and literature. Interestingly, we find that Docetaxel appears in our training set. It is worth noting that Paclitaxel and Docetaxel are similar molecules with the same taxane core. This reflects that our model can utilize drug similarity information to make meaningful predictions. Besides, we also predict the drug–disease associations of AD repositioning candidates in phase 3 clinical trials as of 2021 (Cummings *et al.* 2022). We focus on five drugs: Caffeine, Escitalopram, Guanfacine, Hydralazine and Metformin and their association with AD. Our model predicts the drug–disease associations with the highest median rank compared to the six baseline models (Supplementary Table S1). We can also observe that our model predicts more drugs among the top 100 predictions.

**Table 4.** New candidate drugs ranked by AdaDR prediction scores for Alzheimer’s disease (OMIM:104300) and Breast carcinoma (OMIM:114480).

Diseases	Rank	DrugBank IDs	Candidate drugs	Evidences
AD	1	DB00747	Scopolamine	Nakamura *et al.* (2018)
	2	DB00502	Haloperidol	Devanand *et al.* (2011)
	3	DB00190	Carbidopa	Di Bona *et al.* (2012)
	4	DB00268	Ropinirole	Bertram *et al.* (2007)
	5	DB00387	Procyclidine	Haug *et al.* (2007)
BRCA	1	DB00515	Cisplatin	Daaboul *et al.* (2019)
	2	DB01229	Paclitaxel	Ramaswamy *et al.* (2012)
	3	DB00650	Leucovorin	Lin *et al.* (2007)
	4	DB00773	Etoposide	Polak *et al.* (2017)
	5	DB02546	Vorinostat	Kim *et al.* (2018)

**Figure 7.**
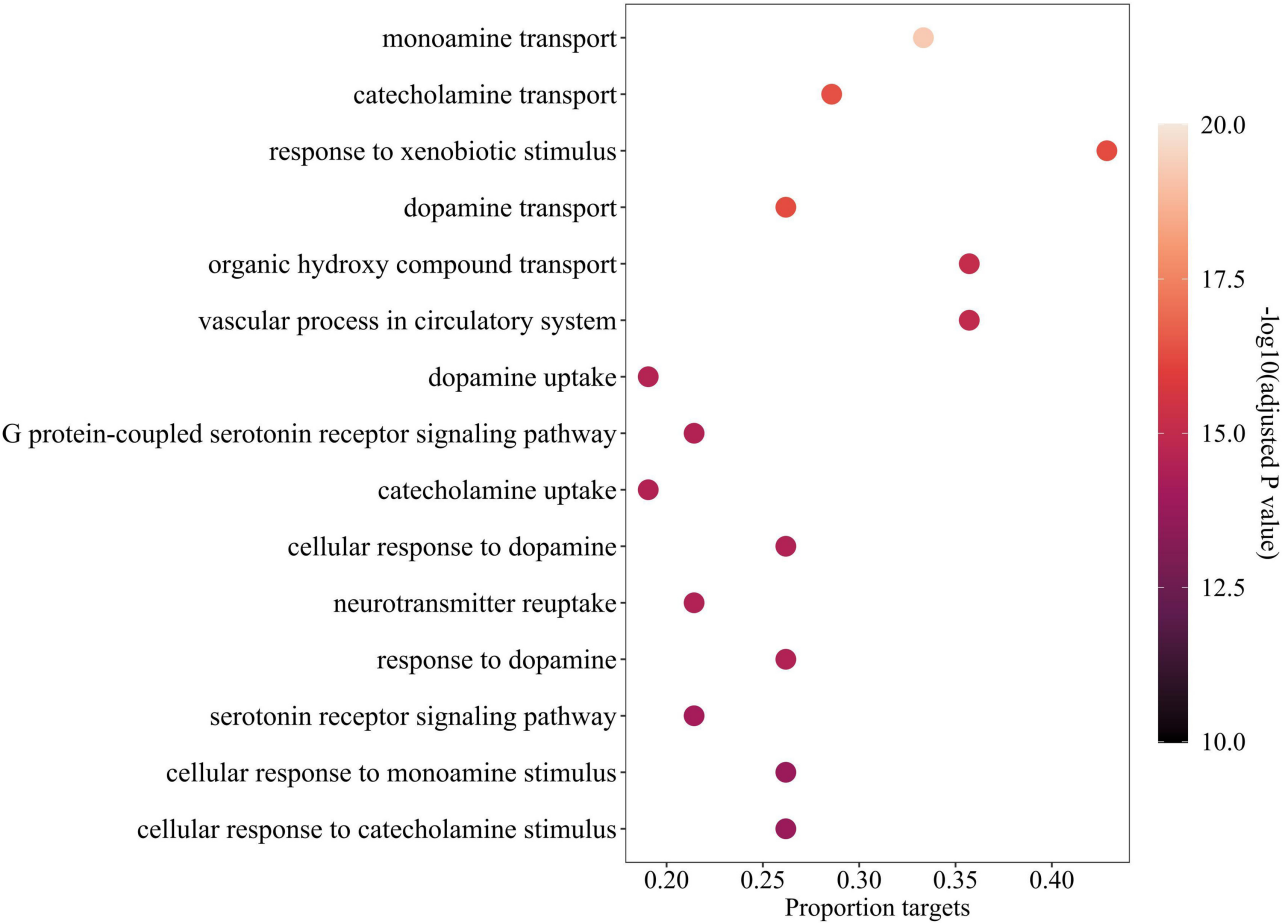
Enriched gene ontology terms (Biological Process) among all predicted AD drug targets. The *x* axis shows the proportion of targets mapped to each pathway.

In addition to the above analysis, we also conduct gene ontology enrichment analysis for the predicted drugs to demonstrate the utility of AdaDR. Taking AD as an example, we collect target information from DrugBank for the predicted top 5 drugs. Then, the Bioconductor package clusterProfiler (Yu *et al.* 2012) is used to perform the gene ontology enrichment analysis. It utilizes the Gene Ontology database (The Gene Ontology Consortium 2019). To better display the potential biology processes related to AD protein targets, we select the top 15 terms based on adjusted *P* value. The result is shown in Fig. 7. Gene ontology enrichment analysis recovers existing mechanisms and also helps identify new processes related to AD protein targets, such as monoamine transport, dopamine uptake and vascular process in circulatory system. The enriched gene ontology categories indicate that predicted AD-related drug targets modulate common regulatory processes. Besides, for biological processes that have not been explored in depth, e.g. serotonin receptor(Geldenhuys and Van der Schyf 2011) and urotransmitter reuptake(Francis 2005), may provide new perspectives for the treatment of AD.

## 4 Conclusion

In this paper, we have proposed AdaDR based on graph neural networks and attention mechanism to model the drug–disease associations in drug repositioning tasks. We integrate the feature space and topology space information and then introduce the consistency constraint to regularize the embeddings in different spaces and propose a simple, efficient, yet effective method AdaDR, which significantly enhanced the performance of drug repositioning tasks. Extensive experiments demonstrated that AdaDR is superior to current prediction methods and various ablation and model studies demystified the working mechanism behind such performance.

Even though AdaDR has achieved better performance, there are still some limitations. First, the integration of multi-dimensional drug and disease data for precision repositioning plays an important role, but AdaDR only uses drug–drug and disease–disease similarity. In future work, we will consider more biological information involved in drug repositioning, such as genes, targets, chemical structures, drug–target interactions and pathways. Second, despite our proposed model can infer new drugs for diseases by using similarity feature, it still lacks the explainability for the predicted result. In the future, we can collect more prior biological knowledge, such as disease phenotypes, drug side effects, disease semantic similarity and so on to construct a knowledge graph network and design an interpretable model.

## Supplementary Material

btad748_Supplementary_Data

## Data Availability

The data underlying this article are available in our provided github repository at https://github.com/xinliangSun/AdaDR.

## References

[btad748-B1] Baker NC , Ekins S, Williams AJ et al A bibliometric review of drug repurposing. Drug Discov Today 2018;23:661–72. 29330123 10.1016/j.drudis.2018.01.018 PMC5963941

[btad748-B2] Berg RVD , Kipf TN, Welling M. Graph convolutional matrix completion. In: *SIGKDD*, 2018.

[btad748-B3] Bertram L , McQueen MB, Mullin K et al Systematic meta-analyses of Alzheimer disease genetic association studies: the AlzGene database. Nat Genet 2007;39:17–23. 17192785 10.1038/ng1934

[btad748-B4] Cai L , Lu C, Xu J et al Drug repositioning based on the heterogeneous information fusion graph convolutional network. Brief Bioinform 2021;22:bbab319. 34378011 10.1093/bib/bbab319

[btad748-B5] Chen H , Cheng F, Li J. idrug: integration of drug repositioning and drug–target prediction via cross-network embedding. PLoS Comput Biol 2020;16:e1008040. 32667925 10.1371/journal.pcbi.1008040 PMC7384678

[btad748-B6] Cummings J , Lee G, Nahed P et al Alzheimer’s disease drug development pipeline: 2022. Alzheimers Dement (N Y) 2022;8:e12295. 35516416 10.1002/trc2.12295 PMC9066743

[btad748-B7] Daaboul HE , Dagher C, Taleb RI et al β-2-Himachalen-6-ol inhibits 4T1 cells-induced metastatic triple negative breast carcinoma in murine model. Chem Biol Interact 2019;309:108703. 31194954 10.1016/j.cbi.2019.06.016

[btad748-B8] Davis AP , Grondin CJ, Johnson RJ et al The comparative toxicogenomics database: update 2017. Nucleic Acids Res 2017;45:D972–8. 27651457 10.1093/nar/gkw838 PMC5210612

[btad748-B9] Davis J , Goadrich M. The relationship between precision–recall and roc curves. In: *Proceedings of the 23rd International Conference on Machine Learning, Pittsburgh, Pennsylvania, USA*, 2006, 233–40.

[btad748-B10] Devanand DP , Pelton GH, Cunqueiro K et al A 6-month, randomized, double-blind, placebo-controlled pilot discontinuation trial following response to haloperidol treatment of psychosis and agitation in alzheimer’s disease. Int J Geriatric Psychiatry 2011;26:937–43. 10.1002/gps.2630 PMC3685500 21845596

[btad748-B11] Di Bona D , Rizzo C, Bonaventura G et al Association between interleukin-10 polymorphisms and Alzheimer’s disease: a systematic review and meta-analysis. J Alzheimers Dis 2012;29:751–9. 22356904 10.3233/JAD-2012-111838

[btad748-B12] Fiscon G , Paci P. SAveRUNNER: an R-based tool for drug repurposing. BMC Bioinformatics 2021;22:150–10. 33757425 10.1186/s12859-021-04076-w PMC7987121

[btad748-B13] Francis PT. The interplay of neurotransmitters in Alzheimer’s disease. CNS Spectrums 2005;10:6–9. 10.1017/s1092852900014164 16273023

[btad748-B14] Geldenhuys WJ , Van der Schyf CJ. Role of serotonin in Alzheimer’s disease: a new therapeutic target? CNS Drugs 2011;25:765–81. 21870888 10.2165/11590190-000000000-00000

[btad748-B15] Gottlieb A , Stein GY, Ruppin E et al Predict: a method for inferring novel drug indications with application to personalized medicine. Mol Syst Biol 2011;7:496. 21654673 10.1038/msb.2011.26 PMC3159979

[btad748-B16] Haug KH , Myhrer T, Fonnum F. The combination of donepezil and procyclidine protects against soman-induced seizures in rats. Toxicol Appl Pharmacol 2007;220:156–63. 17289099 10.1016/j.taap.2006.12.023

[btad748-B17] Iorio F , Bosotti R, Scacheri E et al Discovery of drug mode of action and drug repositioning from transcriptional responses. Proc Natl Acad Sci USA 2010;107:14621–6. 20679242 10.1073/pnas.1000138107 PMC2930479

[btad748-B18] Kim J , Piao H-L, Kim B-J et al Long noncoding RNA MALAT1 suppresses breast cancer metastasis. Nat Genet 2018;50:1705–15. 30349115 10.1038/s41588-018-0252-3 PMC6265076

[btad748-B19] Kipf TN , Welling M. Semi-supervised classification with graph convolutional networks. In: *Proc. ICLR*, 2017, pp. 1–14.

[btad748-B20] Lamb J , Crawford ED, Peck D et al The connectivity map: using gene-expression signatures to connect small molecules, genes, and disease. Science 2006;313:1929–35. 17008526 10.1126/science.1132939

[btad748-B21] Li J , Zhang S, Liu T et al Neural inductive matrix completion with graph convolutional networks for miRNA–disease association prediction. Bioinformatics 2020;36:2538–46. 31904845 10.1093/bioinformatics/btz965

[btad748-B22] Li J , Wang J, Lv H et al IMCHGAN: inductive matrix completion with heterogeneous graph attention networks for drug–target interactions prediction. IEEE/ACM Trans Comput Biol Bioinform 2022;19:655–65. 34115592 10.1109/TCBB.2021.3088614

[btad748-B23] Liang X , Zhang P, Yan L et al LRSSL: predict and interpret drug–disease associations based on data integration using sparse subspace learning. Bioinformatics 2017;33:1187–96. 28096083 10.1093/bioinformatics/btw770

[btad748-B24] Lin C-C , Cheng A-L, Hsu C-H et al A phase II trial of weekly paclitaxel and high-dose 5-fluorouracil plus leucovorin in patients with chemotherapy-pretreated metastatic breast cancer. Anticancer Res 2007;27:641–5. 17348454

[btad748-B25] Luo H , Wang J, Li M et al Drug repositioning based on comprehensive similarity measures and bi-random walk algorithm. Bioinformatics 2016;32:2664–71. 27153662 10.1093/bioinformatics/btw228

[btad748-B26] Luo H , Li M, Yang M et al Biomedical data and computational models for drug repositioning: a comprehensive review. Brief Bioinform 2021;22:1604–19. 32043521 10.1093/bib/bbz176

[btad748-B27] Meng Y , Lu C, Jin M et al A weighted bilinear neural collaborative filtering approach for drug repositioning. Brief Bioinform 2022;23:bbab581. 35039838 10.1093/bib/bbab581

[btad748-B28] Nakamura A , Kaneko N, Villemagne VL et al High performance plasma amyloid-β biomarkers for Alzheimer’s disease. Nature 2018;554:249–54. 29420472 10.1038/nature25456

[btad748-B29] Polak P , Kim J, Braunstein LZ et al A mutational signature reveals alterations underlying deficient homologous recombination repair in breast cancer. Nat Genet 2017;49:1476–86. 28825726 10.1038/ng.3934 PMC7376751

[btad748-B30] Pushpakom S , Iorio F, Eyers PA et al Drug repurposing: progress, challenges and recommendations. Nat Rev Drug Discov 2019;18:41–58. 30310233 10.1038/nrd.2018.168

[btad748-B31] Ramaswamy B , Fiskus W, Cohen B et al Phase I–II study of vorinostat plus paclitaxel and bevacizumab in metastatic breast cancer: evidence for vorinostat-induced tubulin acetylation and hsp90 inhibition in vivo. Breast Cancer Res Treat 2012;132:1063–72. 22200869 10.1007/s10549-011-1928-x PMC3486521

[btad748-B32] Saito T , Rehmsmeier M. The precision–recall plot is more informative than the ROC plot when evaluating binary classifiers on imbalanced datasets. PloS ONE 2015;10:e0118432. 25738806 10.1371/journal.pone.0118432 PMC4349800

[btad748-B33] Sun X , Wang B, Zhang J et al Partner-specific drug repositioning approach based on graph convolutional network. IEEE J Biomed Health Inform 2022;26:5757–65. 35921345 10.1109/JBHI.2022.3194891

[btad748-B34] The Gene Ontology Consortium. The gene ontology resource: 20 years and still going strong. Nucleic Acids Res 2019;47:D330–8. 30395331 10.1093/nar/gky1055 PMC6323945

[btad748-B35] Wang X , Zhu M, Bo D et al AM-GCN: adaptive multi-channel graph convolutional networks. In *Proceedings of the 26th ACM SIGKDD International Conference on Knowledge Discovery & Data Mining, Virtual Event, CA, USA*, 2020, 1243–1253.

[btad748-B36] Wang Y , Aldahdooh J, Hu Y et al DrugRepo: a novel approach to repurposing drugs based on chemical and genomic features. Sci Rep 2022;12:21116–3. 36477604 10.1038/s41598-022-24980-2 PMC9729186

[btad748-B37] Yang M , Luo H, Li Y et al Drug repositioning based on bounded nuclear norm regularization. Bioinformatics 2019;35:i455–63. 31510658 10.1093/bioinformatics/btz331 PMC6612853

[btad748-B38] Yu G , Wang L-G, Han Y et al clusterprofiler: an R package for comparing biological themes among gene clusters. OMICS 2012;16:284–7. 22455463 10.1089/omi.2011.0118 PMC3339379

[btad748-B39] Yu Z , Huang F, Zhao X et al Predicting drug–disease associations through layer attention graph convolutional network. Brief Bioinform 2021;22:bbaa243. 33078832 10.1093/bib/bbaa243

