# Supplementary material for: Drug repositioning with adaptive graph convolutional networks
Source: Bioinformatics. 2023 Dec 9;40(1):btad748. doi: 10.1093/bioinformatics/btad748 (PMC10761094; doi:10.1093/bioinformatics/btad748)
Supplement: btad748_Supplementary_Data [file btad748_supplementary_data.pdf]

Table S1. Predicted rankings of repositioning candidate drugs in phase 3 clinical trial for Alzheimer's disease in 2021. The ranks of the drugs are shown in the table and the highest rank for each drug is highlighted in bold.

Drugs	AdaDR	NIMCGCN	DRWBNCF	DRHGCN	iDrug	BNNR	MBiRW
Caffeine	**41**	71	572	441	580	396	356
Escitalopram	263	531	**66**	508	192	515	362
Guanfacine	301	482	320	**128**	451	434	227
Hydralazine	379	572	304	142	524	189	**80**
Metformin	**64**	218	190	377	195	80	231
Median	**210**	375	290	319	388	322	251
